# Noninvasive Markers of Arteritic Anterior Ischemic Optic Neuropathy in Giant Cell Arteritis

**DOI:** 10.18502/jovr.v17i2.10808

**Published:** 2022-04-29

**Authors:** Vidhi Bajpai, Siddharth Madan, Gunjan Rana

**Affiliations:** ^1^Department of Ophthalmology, Lady Hardinge Medical College & Associated Hospitals, University of Delhi, New Delhi, India; ^2^Department of Ophthalmology, University College of Medical Sciences and Associated GTB Hospital, University of Delhi, New

##  Dear Editor,

**Figure 1:**
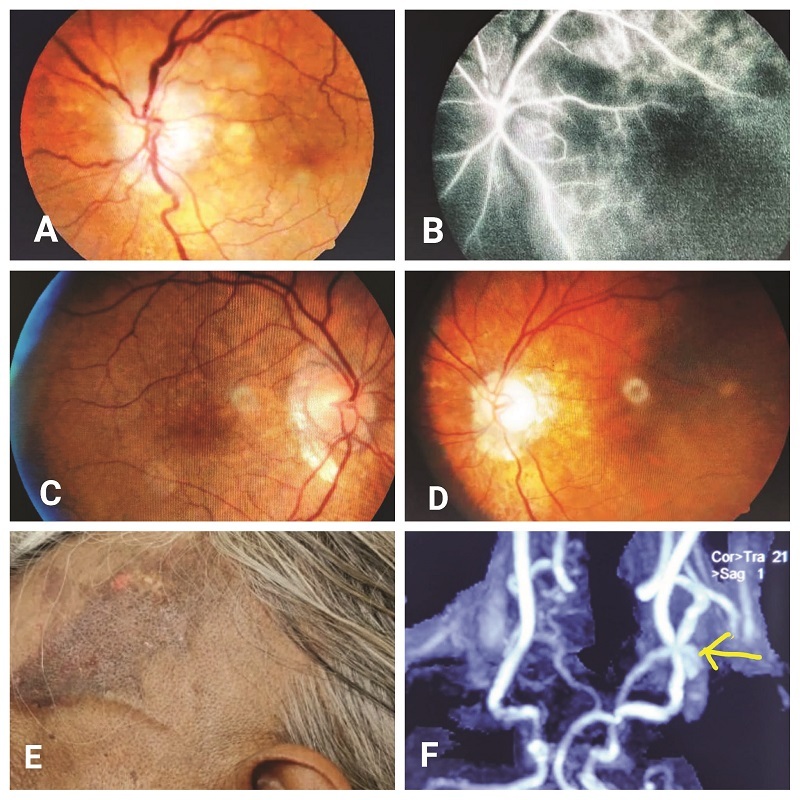
Pale disc edema with venous dilatation at presentation (A). FFA revealed choroidal ischemia with delayed AV transit (B). Right eye was normal (C). Optic disc pallor after 45 days of presentation (D). Left temporal scalp showed purpuric lesion with prominent temporal artery that was biopsied (E). MRA demonstrated ICA narrowing (F).

This is with reference to the recently published article by Aghdam et al.^[1]^ The authors have suggested that giant cell arteritis could be diagnosed on clinical grounds rather than relying on temporal artery biopsy (TAB).^[1]^ While we agree with the authors' conclusions, it also needs a pointer that around 25% of patients of giant cell arteritis (GCA) can present with visual loss alone due to arteritic anterior ischemic optic neuropathy (AAION).^[2]^ Patients who do not comply for proper visual acuity assessment due to old age or an underlying neurocognitive disorder may lead to a delay in diagnosis of AAION. Classical presenting features [Figure 1A–1F] like chalky white optic disc edema, jaw claudication, and scalp tenderness may not always exist.^[2]^ AAION is a rare disease in the Asian population hence a strong suspicion of its presence is required for averting the potentially blinding sequel. In this era of resorting to noninvasive modalities of establishing diagnosis, there is a need to explore such markers in elderly population where GCA-causing AAION is common. Standard fluorescein angiography available in most clinical settings shows choroidal ischemia with delayed filling of the optic disc [Figure 1B]. These changes in optic nerve head may possibly be visualized using a noninvasive technique like optical coherence tomography (OCT) that may show thinning of the retinal nerve fiber layer. OCT-angiography may reveal reductions in vessel density and vessel tortuosity in AAION with worse values than non-arteritic anterior ischemic optic neuropathy (NAION).^[3]^ OCT angiography availability everywhere is an issue yet it currently stands as a noninvasive marker in nearly all retinal, optic nerve head-related disorders in the current times. Further, every patient on presentation undergoes hematological and biochemical testing for systemic evaluation. In a study by Inanc et al, neutrophil lymphocyte ratio (NLR) seemed as a reliable index to differentiate between NAION and AAION. NLR value was found significantly higher in AAION patients in their study.^[4]^ This was also observed in an 80-year-old female [Figure 1A–1E] who presented with classical features of AAION due to GCA and had NLR ratio of 3.84 (normal range, 1.97 ± 0.31).^[4]^ Almost all cases seen in neuro–ophthalmology clinics need imaging. A recent study conducted by Sugihara et al in Japan concluded that large vessel lesions (LVLs), especially those in aorta, correlated with poor treatment outcomes in GCA and early treatment intensity could be determined by their presence or absence.^[5]^ Careful analysis of magnetic resonance angiography revealed attenuated caliber of internal, external, and common carotid artery apart from both temporal arteries [Figure 1F]. These LVLs seem a reliable index for diagnosing GCA. Although TAB remains a part of diagnostic work-up in most settings, the procedure is inconvenient to elderly patients and has potentially high false negative rate in its ability to diagnose GCA.^[6]^ In addition, ability to detect temporal artery on ultrasound and to secure a TAB is not possible in all centers, especially in rural areas.^[3]^It seems logical to investigate for noninvasive markers for diagnoses of GCA in the elderly based on anecdotal reports in literature.

##  Financial Support and Sponsorship

Nil.

##  Conflicts of Interest

There are no conflicts of interest.
